# Effect of S-abscisic acid supplementation on the growth performance and antioxidant status of nursery pigs

**DOI:** 10.1093/tas/txag086

**Published:** 2026-06-24

**Authors:** Ron Aldwin S Navales, Jamil E G Faccin, Joel M DeRouchey, Katelyn N Gaffield, Jordan T Gebhardt, Robert D Goodband, Theresa Rathbun, Jason C Woodworth, Daisuke Matsui, Izuru Shinzato, Tetsuya Hiraoka, Tsuyoshi Tonoue, Mike D Tokach

**Affiliations:** Department of Animal Sciences and Industry, College of Agriculture, Kansas State University, Manhattan, KS, 66506-0201, United States; Department of Animal Sciences and Industry, College of Agriculture, Kansas State University, Manhattan, KS, 66506-0201, United States; Department of Animal Sciences and Industry, College of Agriculture, Kansas State University, Manhattan, KS, 66506-0201, United States; Department of Animal Sciences and Industry, College of Agriculture, Kansas State University, Manhattan, KS, 66506-0201, United States; Department of Diagnostic Medicine/Pathobiology, College of Veterinary Medicine, Kansas State University, Manhattan, KS, 66506-0201, United States; Department of Animal Sciences and Industry, College of Agriculture, Kansas State University, Manhattan, KS, 66506-0201, United States; Department of Animal Sciences and Industry, College of Agriculture, Kansas State University, Manhattan, KS, 66506-0201, United States; Department of Animal Sciences and Industry, College of Agriculture, Kansas State University, Manhattan, KS, 66506-0201, United States; Sumitomo Chemical Co., Ltd., Tokyo, 103-6020, Japan; Sumitomo Chemical Co., Ltd., Tokyo, 103-6020, Japan; Sumitomo Chemical Co., Ltd., Tokyo, 103-6020, Japan; Sumitomo Chemical Co., Ltd., Tokyo, 103-6020, Japan; Department of Animal Sciences and Industry, College of Agriculture, Kansas State University, Manhattan, KS, 66506-0201, United States

**Keywords:** antioxidant, glutathione, nursery pigs, S-abscisic acid

## Abstract

A total of 320 nursery pigs (DNA 241 × 600; initially 5.4 ± 0.52 kg) were used to evaluate the effect of dietary S-abscisic acid (S-ABA) supplementation on the growth performance and antioxidant status of pigs. Pigs were weaned at approximately 18 d of age and assigned to pens in generalized randomized block design with sex and weight category as blocking factors. Pigs were fed a common phase 1 diet for 7 d postweaning. On d 7 post-weaning, pen of pigs (6.4 ± 0.57 kg) within sex × weight blocks were randomly allotted to 1 of 4 dietary treatments. Treatments included a conventional nursery diet (Control) and 3 diets which used the Control formulation with increasing S-ABA (0.5, 1.0 and 5.0 mg/kg). Treatments were provided during phases 2 (d 0 to 14) and 3 (d 14 to 35). Growth performance was measured weekly. Additionally, 32 pigs on d 0 and 2 pigs per pen on d 14 and 35 were bled to assess the erythrocytes’ total glutathione (GSH + GSSG), reduced glutathione (GSH), oxidized to reduced glutathione ratio (GSSG:GSH), and serum total antioxidant capacity (TAC), superoxide dismutase (SOD), and thiobarbituric acid reactive substances (TBARS). At the termination of the trial, 2 pigs in each pen were euthanized and duodenal, jejunal, and ileal mucosa were collected to measure GSH + GSSG, GSH and GSSG:GSH. No interactive effects between S-ABA and sex on growth performance were observed. Additionally, increasing S-ABA did not influence growth performance. There were also no interactions between S-ABA and collection day on antioxidant parameters. Erythrocyte GSH + GSSG tended to increase (linear, *P* = 0.056) with increasing S-ABA in the diet on d 14, while GSH tended to increase (linear, *P* = 0.096) at d 14 and increased up to 1 mg/kg S-ABA (quadratic, *P* = 0.100) on d 35 with little increase thereafter. The d 35 GSSG:GSH ratio decreased (quadratic, *P* = 0.022) with increasing S-ABA in the diet. Serum TAC, SOD and TBARS were not influenced by S-ABA in the diet. Similarly, dietary S-ABA had no effect on GSH + GSSG, GSH and GSSG:GSH in the intestinal mucosa. In conclusion, supplementation of S-ABA in the diet reduced the erythrocyte GSSG:GSH ratio without negative effects on growth performance.

## Teaser

S-abscisic acid is a novel antioxidant that improves antioxidant status of nursery pigs by reducing the oxidized to reduced glutathione ratio in red blood cells.

## Introduction

Weaning is a well-recognized stressor in piglets, and its impact on swine production has been extensively documented. Specifically, weaning-induced stress alters oxidative indicators which may contribute to the development of oxidative stress in pigs (Hong et al. 2024). A variety of nutrient are used to prevent oxidative stress in animals including vitamin E, which protects cell membranes from lipid peroxidation by neutralizing free radicals, and selenium, which plays a crucial role as a component of glutathione peroxidase which reduces hydrogen peroxide (NRC 2012). Additionally, retinol (vitamin A) plays a crucial role in normal growth, reproduction, and vision and also possesses antioxidant properties (Shastak et al. 2023). Retinol scavenges reactive oxygen species, boosts antioxidant enzyme activities, and promotes antioxidant defense mechanisms (Kozlov et al. 2024). While mammals have the potential to synthesize vitamin A from carotenoids that are present in the plant-based ingredients, the efficiency of synthesis is limited.

S-abscisic acid (S-ABA) is a direct derivative of carotenoid and shares a structural similarity with retinoid, which is known for its antioxidant properties. S-abscisic acid is an important phytohormone that regulates many aspects of plant growth and development including embryo maturation, seed dormancy, germination, cell division and elongation, floral induction, and responses to environmental stresses (Finkelstein 2013). In animals, it has been demonstrated that S-ABA plays a key role in resistance against different microbial pathogens, inflammatory response, and act as a potent antioxidant molecule (Baliño et al. 2019). Soti et al. (2019) reported that S-ABA was able to reduce oxidative stress in rat brains as evident with reduced malondialdehyde (MDA) concentration, reduced H_2_O_2_ levels in rat diencephalon, and increased antioxidant enzyme catalase and peroxidase activities.

Several biological tests can be used to assess the antioxidant status of an organism. For example, serum total antioxidant capacity (TAC), superoxide dismutase (SOD), and thiobarbituric acid reactive substances (TBARS) can be used as criteria to evaluate the antioxidant status of an organism. Total antioxidant capacity measures the cumulative action of all the antioxidants present in plasma and body fluids and provides information on the balance between oxidants and antioxidants, thus the redox status (Ghiselli et al. 2000). Superoxide dismutase is a metalloenzyme that catalyzes the dismutation of superoxide anion to molecular oxygen and peroxide and thus is an important part of the cellular antioxidant defense mechanism (Le et al. 2023). Malondialdehyde (MDA) is a lipid peroxidation product that reacts with thiobarbituric acid to form TBARS. Thus, TBARS is a further indicator of the level of oxidative stress within biological samples (Aguilar Diaz De Leon and Borges 2020). Additionally, the levels of total glutathione (GSH + GSSG), reduced glutathione (GSH), and ratio of oxidized and reduced glutathione (GSSG:GSH) in erythrocytes and intestinal mucosa can be used. Glutathione is the main antioxidant that works in the detoxification of electrophiles and alleviation of oxidative stress caused by reactive oxygen species (ROS). As a reducing agent, GSH reduces ROS and is converted to GSSG. Therefore, GSSG:GSH reflects the redox status of the cells and serves as a sensitive indicator of oxidative stress (Hou et al. 2023).

Limited data exists evaluating S-ABA supplementation in swine diets. Thus, this study aimed to evaluate the effect of dietary S-ABA supplementation on the growth performance and antioxidant status of nursery pigs using erythrocyte and intestinal mucosa glutathione levels and serum TAC, SOD, and TBARS levels as measures of antioxidant status.

## Materials and methods

This experiment followed the guidelines set by the Kansas State University Institutional Animal Care and Use Committee (IACUC # 4942). The study was conducted at the Kansas State University Swine Teaching and Research Center in Manhattan, KS. The facility was completely enclosed and environmentally controlled. Pigs were placed in 1.22 m × 1.22 m pens that contained a four-hole dry self-feeder (Thorp Equipment, Thorp, WI) and a nipple drinker for ad libitum access to feed and water.

### Animals and diets

A total of 320 pigs (DNA 241 × 600; DNA Genetics, Columbus NE) with initial body weight (BW) of 5.4 ± 0.52 kg were weaned at approximately 18 d of age. Pigs were assigned to pens in generalized randomized block design. Pigs were fed a common phase 1 diet [NE: 2580 kcal/kg and standardized ileal digestible (SID) Lys: 1.50%] for 7 d postweaning. On d 7 post-placement (considered d 0 of the study), pens of pigs (BW: 6.4 ± 0.57 kg) within sex and weight blocks were randomly allotted to one of four dietary treatments. Each pen had 5 pigs and there were 16 pens per treatment (2 pens per sex in each weight category). Treatments included a conventional nursery diet (Control), and 3 diets based on the Control formulation with increasing S-ABA (0.5, 1.0, and 5.0 mg/kg S-ABA). The experimental period included phase 2 (d 0 to 14) and phase 3 (d 14 to 35) periods. For each phase, dietary treatments were derived from a single corn-soybean meal-specialty ingredient-based diet and three premixes that contained 0.1, 0.2, and 1.0% S-ABA were incorporated into the feed at an inclusion rate of 0.05% to provide 0.5, 1.0, and 5.0 mg/kg dietary S-ABA, respectively. The S-ABA premixes were provided by Sumitomo Chemical Co. Ltd. (Tokyo, Japan) and were composed of S-ABA and limestone as an inert carrier. Internal analysis conducted by Sumitomo Chemical Co. Ltd. showed that the premix contained 0.107, 0.196, and 1.009% S-ABA. In place of the S-ABA premix, 0.05% ground corn was added to the Control diet. All nutrients were set to meet or exceed nutrient recommendations established by the Nutrient Requirements of Swine (2012, Table 1).

**Table 1.** Composition, calculated analysis, and analyzed composition of experimental diets (as-fed basis)^a^

Item	Phase 1	Phase 2	Phase 3
**Ingredient, %**			
** Corn**	44.90	55.70	66.50
** Soybean meal, 47.7% CP**	21.30	25.80	29.70
** Blood plasma**	2.50	—	—
** Specialty soy product^b^**	6.00	3.75	—
** Whey powder**	20.00	10.0	—
** Soybean oil**	2.00	1.00	—
** Calcium carbonate**	0.60	0.60	0.80
** Monocalcium phosphate, 21% P**	0.60	0.90	1.00
** Salt**	0.30	0.60	0.60
** L-Lys-HCl**	0.45	0.47	0.50
** DL-Met**	0.28	0.23	0.16
** L-Thr**	0.17	0.18	0.20
** L-Trp**	0.03	0.05	0.04
** L-Val**	0.13	0.12	0.13
** Trace mineral premix^c^**	0.15	0.15	0.15
** Zinc oxide^c^**	0.40	0.26	—
** Vitamin premix^d^**	0.125	0.125	0.125
** Vitamin A^e^**	0.00004	0.00004	—
** Vitamin E^f^**	0.00101	0.00101	—
** Phytase^g^**	0.08	0.08	0.08
** S-ABA^h^^,^^i^**	—	+/−	+/−
**Calculated analysis**			
** SID AA^j^, %**			
** Lys**	1.50	1.35	1.30
** Ile: Lys**	56	57	55
** Leu: Lys**	111	114	116
** Met: Lys**	38	37	33
** Met + Cys: Lys**	60	58	54
** Thr: Lys**	62	62	62
** Trp: Lys**	19	20	19
** Val: Lys**	70	70	70
** SID Lys: NE, g/Mcal**	5.83	5.41	5.34
** NE, kcal/kg**	2575	2495	2430
** STTD P, %**	0.40	0.39	0.35
** Vitamin A, IU/kg**	2205	2205	1764
** Vitamin E, IU/kg**	16.1	16.1	11.0
** S-ABA, mg/kg**	—	0 to 5.0	0 to 5.0
**Chemical analysis^k^**			
** Moisture, %**	—	11.8	12.6
** Dry matter, %**	—	88.2	87.4
** Crude protein, %**	—	21.3	20.6
** Ether extract, %**	—	3.4	2.7
** Crude fiber, %**	—	1.7	2.0
** Ash, %**	—	5.4	4.4
** Total Ca, %**	—	0.74	0.68
** Total P, %**	—	0.62	0.53
** S-ABA, mg/kg^l^**			
** Control**	—	—	—
** Control + 0.5 mg/kg S-ABA**	—	0.66	0.64
** Control + 1.0 mg/kg S-ABA**	—	1.21	1.14
** Control + 5.0 mg/kg S-ABA**	—	5.65	5.36

a Phase 1 was fed from d 0 to 7 post-weaning. Phases 2 and 3 were fed from d 7 to 21 and d 21 to 42 post-weaning, respectively (or d 0 to 14 and d 14 to 35 of experimental period).

b HP 300, Hamlet Protein, Findlay, OH.

c Provided the following levels in the diet: 110 mg/kg Zn, 33 mg/kg Mn, 17 mg/kg Cu, 0.30 mg/kg I, 0.30 mg/kg Se. Provided an additional 3000 and 2000 mg/kg added Zn in Phase 1 and 2, respectively.

d Provided per kg diet: 1764 IU Vit. A, 1654 IU Vit. D, 11.0 IU Vit. E, 3.31 mg Vit. K, 33.1 mcg Vit. B12, 49.6 mg Niacin, 27.6 mg Pantothenic acid, 8.27 mg Riboflavin.

e Vitamin A (as retinyl acetate) was added to Phases 1 and 2, resulting to 2205 IU/kg total Vit. A.

f Vitamin. E (as dl-α-tocopherol acetate) was added to Phases 1 and 2, resulting in 16.1 IU/kg total Vit. E.

g Ronozyme HiPhos 2700 (DSM Nutritional Products, Parsippany, NJ) provided an estimated release of 0.13% STTD P with 2160 FYT/kg.

h Sumitomo Chemical Co., Ltd. Tokyo, Japan. Three premixes containing 0.1, 0.2 and 1.0% S-abscisic acid (S-ABA) were added to the basal diet at 0.05% resulting in dietary S-ABA of 0.5, 1.0 and 5.0 mg/kg.

i Internal analysis of Sumitomo Chemical Co., Ltd. showed that the premixes contained 0.107, 0.196 and 1.009% S-ABA.

j Standardized ileal digestible amino acid.

k Basal phase 2 and 3 diets were analyzed in duplicate for moisture, CP, EE, CF, ash, total Ca and total P at Midwest Laboratories, Omaha, NE. Experimental diets were analyzed in triplicate for S-ABA content at Valent BioSciences, Libertyville, IL.

l S-ABA levels were calculated by subtracting the S-ABA level of the control diet with the detected S-ABA of the fortified diets.

Basal and experimental diets were manufactured in meal form at O. H. Kruse Feed Technology Innovation Center at Kansas State University. During bagging, representative samples of the diets were collected using a sampling probe from every fourth bag, pooled, ground and stored at -20°C until analysis. Phase 2 and 3 basal diets were analyzed in duplicate for nutrient content at Midwest Laboratories, Omaha, NE. Analyses included moisture content (MC, AOAC Official Method 930.15, 2005), crude protein (CP; AOAC Official Method 990.03, 2006), ether extract (EE, AOAC Official Method 2003.05, 2006), crude fiber (CF; AOCS Ba 6a-05), ash (AOAC Official Method 942.05, 2006), and total Ca and P (AOAC Official Method 985.01, 2006). Additionally, experimental diets were analyzed in triplicate for S-ABA concentration (ABA LC-MS/MS Method) at Valent BioSciences, Libertyville, IL.

### Sampling, measurements and analytical methods

Throughout the study, pig and feeder weights were measured every 6 to 7 d to determine average daily gain (ADG), average daily feed intake (ADFI), and gain to feed ratio (G:F). Pigs were weighed individually on a platform scale during phase 2 and as a pen during Phase 3. Feed intake, calculated on pen basis, was determined throughout the study as the total amount of feed provided minus feed refused and feed removed. Feed efficiency was calculated as gain to feed.

To evaluate the antioxidant status of pigs, key parameters: GSH + GSSG, GSH, and the GSSG:GSH in erythrocytes, along with serum TAC, SOD, and TBARS were measured. On d 0, blood samples were collected from 32 median-weight pigs, with one pig of each sex per weight category in each treatment group. On d 14, blood samples were taken from two median-weight pigs per pen, and the same pigs were bled again at the end of the study (half on d 34 and the other half on d 35).

For erythrocyte analysis, whole blood samples were collected using EDTA-containing tubes [Coviden Monoject blood collection tube, silicone coated lavender stopper, EDTA (K3) 0.07 mL 15.0% solution, 7 mL draw; Medtronic, Minneapolis, MN]. Samples were centrifuged at 1000 × *g* for 10 min at 0°C, and the erythrocyte fraction (0.5 mL) was lysed in 2 mL of glutathione buffer and kept on ice for 10 min. Samples were deproteinized by adding 1 mL of trichloroacetic acid (TCA) and centrifuged at 4000 × *g* for 15 min at 0°C. The supernatant was then transferred to a fresh tube and stored at −80°C until analysis. For serum analysis, whole blood samples were collected in tubes without additives (Coviden Monoject blood collection tube, silicone coated red stopper, no additive, 7 mL draw; Medtronic, Minneaolis, MN). After clotting for at least 30 min, samples were centrifuged at 1500 × *g* for 30 min at 0°C, and the serum supernatant was divided into two polypropylene tubes (PR1MA microcentrifuge tubes, natural boil-proof; Midwest Scientific, St. Louis, MO) and stored at −80°C until analysis.

The levels of GSH + GSSG and GSH were determined using a commercial glutathione quantification kit (Ab205811 GSH/GSSG ratio detection kit II (fluorometric–green); Abcam Inc., Waltham, MA). This assay utilized a proprietary non-fluorescent, water-soluble dye that becomes fluorescent upon reacting with GSH, with fluorescence read using a microplate reader (Synergy HT Multi-Mode Microplate Reader; BioTek Instruments, Inc, Winooski, VT) at Ex/Em = 490/520 nm. Oxidized glutathione (GSSG) was calculated as: (GSH + GSSG—GSH) ÷ 2, reflecting the conversion of GSSG to GSH at a 1:2 ratio.

Serum TAC and SOD levels were analyzed using commercial kits [OxiSelect Total Antioxidant Capacity Assay Kit (STA-360); Cell Biolabs, Inc; San Diego, CA, and Superoxide Dismutase Assay Kit (706002); Cayman Chemical; Ann Arbor, MI, respectively]. The TAC assay measured the reduction of Cu (II) to Cu (I) by antioxidants, with the resulting color measured at 490 nm and compared to a uric acid standard curve. The SOD assay utilized tetrazolium salt to detect superoxide radicals generated by xanthine oxidase and hypoxanthine, defining one unit of SOD as the amount of enzyme required for 50% dismutation of superoxide radicals, measured by the change in absorbance per minute at 25°C and pH 8.0. Thiobarbituric acid reactive substances levels were assessed following a procedure adapted from Aguilar Diaz De Leon and Borges (2020). Briefly, the assay involved the reaction of lipid peroxidation products, primarily MDA, with TBA which led to the formation of TBARS.

Pigs that were bled at the termination of the study were also euthanized for the collection of intestinal mucosae from the duodenum, jejunum, and ileum. Due to the time and labor involved, sample collections were conducted over two days, with 32 pens (one pen per treatment for each sex and weight category) sampled on d 34 and 35 of the study. Following euthanasia, a necropsy was performed to locate the key abdominal landmarks including the pyloris and cecum. Following identification of the small intestine segments, a 7.6 cm section was excised, and the intestine sample was opened, and the mucosa was gently washed with saline solution, then scraped into 10 mL tubes and immediately frozen in dry ice, storing them at −80°C until preparation. For sample preparation, a 100 mg tissue sample was homogenized in 0.4 mL of glutathione buffer. To this homogenate, 100 µL of TCA was added, followed by vortexing and centrifugation at 4000 × *g* for 15 min. The resulting supernatant was transferred to a fresh tube for use in the glutathione assay. The glutathione assays for mucosa samples were the same as to those of erythrocytes.

### Statistical analysis

Statistical analyses were performed using SAS v.9.4 (SAS Institute, Inc., Cary, NC). Pen was used as the experimental unit for the analysis of growth performance, while pig was used as the experimental unit for criteria where multiple pigs within a pen were sampled such as blood and intestinal mucosa parameters. For growth performance and intestinal mucosa glutathione criteria, S-ABA, sex and its interaction were used as fixed effects and weight category (and pen for intestinal mucosa) was used as a random intercept. For erythrocyte glutathione parameters and serum TAC, SOD and TBARS, a constrained longitudinal model was fit following procedures described by Coffman et al. (2016) where the statistical model included the S-ABA × day interaction, day, and sex as fixed effects. Weight category and pen were included as random intercepts, and repeated measures were modelled using an unstructured covariance matrix structure with individual pig as subject. Interaction of sex with other fixed effects were initially modelled and found to be not significant (*p* > 0.10) and therefore was removed from the model. Means were estimated using LSMEANS function with slice option to evaluate differences within each day of collection.

For all criteria measured, linear and quadratic contrasts were evaluated to determine the response to increasing concentration of S-ABA. Probability values less than 0.05 were considered statistically significant, and values between 0.05 and 0.10 were considered tendencies.

## Results and discussion

Oxidative stress can arise from an imbalance between oxidants and antioxidants, often triggered by excessive production of reactive oxygen species (ROS). When ROS levels surpass the capacity of the body’s enzymatic and non-enzymatic antioxidant defenses, it can lead to metabolic disorders and placental dysfunction in sows, as well as impaired growth performance in piglets (Hong et al. 2024). In nursery pigs, oxidative stress can result from a variety of factors, including physiological challenges such as weaning stress and environmental conditions like heat or cold stress. Dietary factors such as mycotoxins, or dietary lipid peroxidative induced stress may also contribute. As a result, a variety of nutrients are used to prevent oxidative stress in animals including vitamin E, selenium, and functional amino acids (Hong et al. 2024; Liang et al. 2023; Rao et al. 2023). Besides nutritional strategies, increasing attention has been given to non-nutritional bioactive compounds in plants, such as polyphenols, plant extracts, and phytohormones, which possess antioxidant and anti-inflammatory properties (Corino and Rossi 2021; Hong et al. 2024). These plant compounds acts as antioxidants by scavenging free radicals, preventing their formation, and inhibiting oxidative stress, and this is achieved through hydrogen atom transfer, single electron transfer, or transition metal chelation (Lang et al. 2024).

S-abscisic acid is a promising candidate phytohormone with antioxidant property. It is an important phytohormone that regulates many aspects of plant growth and development including embryo maturation, seed dormancy, germination, cell division and elongation, floral induction, and responses to environmental stresses (Finkelstein 2013). S-abscisic acid is biosynthesized from C_40_ carotenoids, xanthophylls. Briefly, the process involves the oxidative cleavage of xanthophyll to produce xanthotin, which is then converted to abscisic aldehyde and subsequently oxidized to form S-ABA. The antioxidant properties of S-ABA have been well demonstrated in plants (Li et al. 2022; Nguyen et al. 2023). Under stress condition, S-ABA activates a strong antioxidant defense response in plants, enhancing the activity of key enzymes such as SOD, catalase, peroxidase, and glutathione reductase.

To the best of current knowledge, this experiment is the first to evaluate effect of S-ABA on growth performance and antioxidant status of nursery pigs. Multiple methods were employed to assess the antioxidant status of the pigs to ensure a robust evaluation.

To ensure that the experimental diets contained the desired levels of S-ABA, the chemical compositions of the basal and experimental diets were analyzed and confirmed to match the formulated values before the experiment was initiated (Table 1). For the experimental diets, the analyzed S-ABA levels were higher than the formulated levels but followed an increasing trend. The 0.5, 1.0 and 5.0 mg/kg S-ABA diets had 0.66, 1.21, and 5.65 mg/kg S-ABA in phase 2 and 0.64, 1.14, and 5.36 mg/kg S-ABA in phase 3 diet, respectively. Although not directly analyzed, it is important to note that the levels of vitamins A and E, both known for their antioxidant properties, were formulated to meet the NRC (2012) requirement estimates in all experimental diets. This approach ensured that any observed effects could be attributed to S-ABA supplementation rather than variations in baseline antioxidant nutrients. Moreover, it should be emphasized that S-ABA does not substitute for the distinct biological roles of vitamins A and E. Furthermore, formulating a single basal diet for each of phases 2 and 3 ensured uniformity in the levels of nutrient or compounds with potential antioxidant properties (eg carotenoids in yellow corn) across treatments.

For growth performance, no interaction between S-ABA and sex was observed throughout the study (Table 2). Similarly, S-ABA had no significant effect on growth performance at any time point. However, from d 14 to 21, there was a tendency for increased (quadratic, *p* = 0.053) G:F with increasing dietary S-ABA levels. The lack or minimal effect of antioxidant supplementation on growth performance observed in the present study may suggest that the pigs had an adequate antioxidant status or minimal oxidative stress challenge. In the current trial, one pig was removed due to *Streptococcus* infection, and another was euthanized due to an abdominal hernia, suggesting that the overall health status of the pigs was good and that they were not subjected to significant physiological stressors.

**Table 2.** Effect of S-abscisic acid (S-ABA) on growth performance of nursery barrows and gilts^a^^,^^b^

	S-ABA, mg/kg				SEM	*P* ^c^ =	
	0	0.5	1.0	5.0		Linear	Quadratic
**Body weight, kg**							
** d 0**	6.4	6.5	6.4	6.4	0.56	0.918	0.936
** d 7**	8.7	8.8	8.6	8.7	0.69	0.707	0.617
** d 14**	11.7	12.1	11.6	11.9	0.86	0.467	0.761
** d 21**	14.4	14.9	14.5	14.8	1.05	0.429	0.769
** d 28**	18.9	19.1	18.8	19.3	1.19	0.348	0.700
** d 35**	24.2	24.5	24.0	24.6	1.35	0.393	0.680
**D 0 to 7**							
** ADG, kg**	0.32	0.33	0.31	0.33	0.022	0.495	0.486
** ADFI, kg**	0.40	0.42	0.38	0.42	0.024	0.222	0.133
** G:F, g/kg**	776.1	787.8	801.7	775.1	0.018	0.641	0.263
**D 7 to 14**							
** ADG, kg**	0.43	0.47	0.43	0.46	0.027	0.368	0.977
** ADFI, kg**	0.64	0.67	0.64	0.66	0.038	0.773	0.777
** G:F, g/kg**	670.7	700.0	677.0	697.2	0.012	0.284	0.777
**D 14 to 21**							
** ADG, kg**	0.39	0.40	0.41	0.41	0.030	0.624	0.248
** ADFI, kg**	0.66	0.68	0.66	0.68	0.040	0.438	0.996
** G:F, g/kg**	585.1	586.3	621.4	589.6	0.015	0.864	0.053
**D 21 to 28**							
** ADG, kg**	0.65	0.61	0.62	0.64	0.030	0.825	0.182
** ADFI, kg**	0.86	0.84	0.84	0.87	0.041	0.485	0.475
** G:F, g/kg**	761.3	722.5	736.1	732.3	0.017	0.443	0.165
**D 28 to 35**							
** ADG, kg**	0.80	0.83	0.80	0.82	0.030	0.743	0.764
** ADFI, kg**	1.17	1.18	1.14	1.17	0.048	0.987	0.282
** G:F, g/kg**	686.2	703.2	702.6	700.3	0.012	0.629	0.236
**D 0 to 35**							
** ADG, kg**	0.51	0.52	0.51	0.52	0.025	0.478	0.690
** ADFI, kg**	0.74	0.75	0.73	0.75	0.036	0.530	0.396
** G:F, g/kg**	693.0	694.9	702.0	696.2	0.006	0.902	0.274

a A total of 320 pigs were used in 7-d acclimatization and 35-d experimental feeding trial, with 5 pigs per pen and 16 replicate pens per treatment (2 pens per sex in each weight category). Experimental diets were provided starting 7 d post weaning, which was considered d 0 of the study.

b ADG, average daily gain; ADFI, average daily feed intake; G:F, gain to feed ratio.

c For the analysis, pen was considered the experimental unit, with diet (S-ABA), sex and S-ABA × sex interaction as fixed effects and weight group as a random effect. No interactive effect of diet and sex were observed (*p* > 0.05).

In animals, the impacts of antioxidants are more pronounced in immune response and oxidative stress biomarkers than growth performance. For example, it has been demonstrated that S-ABA plays a key role in resistance against different microbial pathogens, on inflammatory response, and as a potent antioxidant molecule in vitro and in mice (Baliño et al. 2019). The antioxidant role of S-ABA in animals and human medicine has been demonstrated in few studies. For instance, Soti et al. (2019) reported that S-ABA reduced oxidative stress in rat brains, as evidenced by decreased MDA levels, lower H_2_O_2_ concentrations in the diencephalon, and increased activities of antioxidant enzymes such as catalase and peroxidase. Furthermore, Rafiepour et al. (2019) and Shabani et al. (2023) demonstrated the neuroprotective effects of S-ABA against 6-hydroxydopamine (6-OHDA)-induced neurotoxicity in both in vitro and in vivo models of Parkinson’s disease. These protective effects were attributed, at least in part, to S-ABA’s ability to indirectly reduce ROS levels by activating the nuclear receptor peroxisome proliferator-activated receptor gamma (PPARγ), which in turn decreases ROS production and enhances the expression of antioxidant enzymes. The antioxidant potential of S-ABA may also be partly explained by its structural similarity to retinoic acid, a well-known antioxidant compound. Both molecules possess a hydrophobic isoprenoid chain and a terminal carboxylic acid group, enabling them to interact with lipid membranes and bind to nuclear receptors such as PPARγ, thereby modulating gene expression involved in oxidative stress responses.

Several biological tests can be used to assess the antioxidant status of an organism. This includes TAC, SOD, TBARS, and GSSG:GSH (Aguilar Diaz De Leon and Borges 2020; Ghiselli et al. 2000; Hou et al. 2023; Le et al. 2023), among others. Furthermore, oxidative status can be measured on a variety of sample types to give an indication of systemic or localized oxidative status. In the present study, several assays were used to assess serum and mucosal oxidative status. For erythrocyte GSH + GSSG, GSH, and GSSG:GSH, and serum TAC, SOD, and TBARS, no interactive effect of S-ABA and day of collection was observed (Table 3). Similarly, serum TAC, SOD, and TBARS levels were not affected by the dietary inclusion of S-ABA. In contrast, increased vitamin E and polyphenol supplementation in pigs resulted to increased TAC and SOD in the studies of Silva-Guillen et al. (2020) and Rao et al. (2023), respectively. However consistent with the current study, Rao et al. (2023) reported no significant effect on TBARS with increasing vitamin E equivalence. The lack of response in TBARS, TAC, and SOD may be because of the mode of action of S-ABA. As described by Rafiepour et al. (2019) and Shabani et al. (2023), S-ABA exerts its antioxidant effects indirectly by activating the nuclear receptor PPARγ, which subsequently reduces ROS production and upregulates the expression of antioxidant enzymes. In the present study, the absence of changes in TBARS indicates that the animals were not experiencing significant lipid peroxidation. Consequently, the need for S-ABA to activate PPARγ and induce antioxidant enzyme expression may have been minimal under the conditions of this study.

**Table 3.** Effect of S-abscisic acid (S-ABA) on nursery pigs’ erythrocyte GSH + GSSG, GSH and GSSG:GSH and serum TAC, SOD and TBARS^a^^,^^b^

	S-ABA, mg/kg				SEM	*P* ^c^ ^,^ ^d^=		
	0	0.5	1.0	5.0		Day	Diet	
							Linear	Quadratic
**Erythrocytes**								
** GSH + GSSG, nmol/mL**								
** Baseline**	54.36				—	< 0.001	—	—
** d 14**	72.25	71.35	76.91	79.35	4.060		0.056	0.455
** d 35**	41.26	44.26	47.51	49.22	4.129		0.348	0.440
** GSH, nmol/mL**								
** Baseline**	39.39				—	< 0.001	—	—
** d 14**	61.12	59.62	63.58	65.90	3.297		0.096	0.720
** d 35**	32.11	36.06	40.74	40.84	3.911		0.130	0.100
** GSSG:GSH**								
** Baseline**	0.187				—	< 0.001	—	—
** d 14**	0.101	0.103	0.107	0.109	0.0154		0.618	0.787
** d 35**	0.274	0.191	0.130	0.166			0.298	0.022
**Serum**								
** TAC, mmol—CRE**								
** Baseline**	565.8				—	< 0.001	—	—
** d 14**	545.8	568.3	558.2	577.1	32.56		0.312	0.725
** d 35**	627.6	636.1	611.9	645.9	32.37		0.380	0.454
** SOD, U/mL**								
** Baseline**	2.49				—	< 0.001	—	—
** d 14**	3.91	3.66	3.55	3.88	0.317		0.669	0.243
** d 35**	4.58	4.26	4.17	4.31	0.345		0.805	0.294
** TBARS, µmol MDA**								
** Baseline**	7.55				—	0.180	—	—
** d 14**	7.48	7.85	7.52	7.47	0.337		0.730	0.828
** d 35**	7.01	6.94	7.25	7.39	0.331		0.354	0.748

a Blood samples were collected from 32-median weight pigs at d 0 of the experiment to represent baseline levels for the different antioxidant parameters. The 32 pigs were balanced across 4 treatments within each sex × weight category blocks. On d 14, blood samples were taken from two median-weight pigs per pen, and the same pigs were bled again at the end of the study, resulting in 32 observations per treatment.

b GSH+GSSG, total glutathione; GSH, reduced glutathione; GSSG, oxidized glutathione; TAC, total antioxidant capacity; expressed as mmol Cu-reducing equivalent; SOD, superoxide dismutase, expressed as enzyme activity (U) per mL; TBARS, thiobarbituric reactive substance, expressed as µmol malondialdehyde.

c A constrained longitudinal model was fitted with diet (S-ABA) × collection day, day of collection and sex as fixed effects, and weight category and pen as random effects. Repeated measures were modelled using an unstructured covariance matrix structure with individual pig as subject.

d No interactive effect of sex and day with other fixed effects were observed (*p* > 0.05).

**Table 4.** Effect of S-abscisic acid (S-ABA) on duodenal, jejunal and ileal GSH + GSSG, GSH, and GSSG:GSH of nursery barrow and gilts^a^^,^^b^

	S-ABA, mg/kg				SEM	*P* ^c^ =	
	0	0.5	1.0	5.0		Linear	Quadratic
**Duodenum**							
** GSH + GSSG, µmol/g**	1.30	1.24	1.21	1.25	0.055	0.705	0.119
** GSH, µmol/g**	1.10	1.03	0.99	1.02	0.043	0.452	0.053
** GSSG:GSH**	0.09	0.10	0.12	0.11	0.010	0.412	0.140
**Jejunum**							
** GSH + GSSG, µmol/g**	0.98	1.06	1.00	1.08	0.068	0.269	0.868
** GSH, µmol/g**	0.85	0.91	0.85	0.94	0.067	0.242	0.945
**GSSG:GSH**	0.08	0.08	0.10	0.08	0.010	0.839	0.389
**Ileum**							
** GSH + GSSG, µmol/g**	0.65	0.63	0.66	0.63	0.054	0.705	0.879
** GSH, µmol/g**	0.59	0.57	0.58	0.55	0.048	0.476	0.867
** GSSG:GSH**	0.06	0.06	0.08	0.08	0.020	0.210	0.543

a Values represent the mean of 32 observations per treatment. Two median-weight pigs in each pen (same pigs that were bled) were euthanized for the collection of intestinal mucosae from duodenum, jejunum and ileum.

b Levels of GSH + GSSG, GSH and GSSG:GSH are different among sections of intestine (*p* < 0.001).

c For the analysis, pen was considered as experimental unit and pig as observational unit, with diet (S-ABA), sex and S-ABA × sex interactions as fixed effect and weight group and pen as random effects. No interactive effect of diet and sex and main effect of sex were observed (*p* > 0.05).

On the other hand, erythrocyte GSH + GSSG tended to increase (linear, *p* = 0.056) with increasing S-ABA in the diet at d 14 of the trial but not on d 35. Moreover, erythrocyte GSH tended to increase with increasing S-ABA in the diet at d 14 (linear, *p* = 0.096) and d 35 (quadratic, *p* = 0.100). Similar to S-ABA, dietary GSH supplementation has been shown to enhance serum antioxidant capacity, as evidenced by increased GSH levels in nursery pigs subjected to diquat-induced oxidative stress (Liang et al. 2023). As the primary intracellular antioxidant, glutathione plays a crucial role in detoxifying electrophiles and mitigating oxidative stress caused by ROS. Therefore, while S-ABA did not increase the antioxidant enzyme expression in this study, its ability to increase glutathione levels suggests a contributory role in enhancing cellular antioxidant defenses. Additionally, the erythrocyte GSSG:GSH which reflects the redox status of the cells decreased (quadratic, *p* = 0.022) with increasing S-ABA in the diet at d 35. These findings underscore GSSG:GSH as a sensitive and reliable biomarker of oxidative stress, as previously emphasized by Giustarini et al. (2016).

The day of collection also influenced the level of erythrocyte and serum antioxidant parameters. Serum TAC and SOD were greater (*p* < 0.001) at d 35 compared to d 14 of collection and this is consistent with the finding of Silva-Guillen et al. (2020) with vitamin E and polyphenols. This may indicate an increased antioxidant capacity as the pig grew reflecting increased feed consumption. In contrast, erythrocyte GSH + GSSG and GSH were greater (*p* < 0.001) on d 14 compared to d 35 of collection.

In addition to the erythrocytes and serum, the current study also evaluated the glutathione levels in the intestinal mucosa. Except for the tendency of reduced (*p* = 0.054) GSH in the duodenal mucosa with increasing S-ABA in the diet, the levels of GSH + GSSG, GSH, and GSSG:GSH in the duodenal, jejunal, and ileal mucosa were not influenced by the level of S-ABA in the diet (Table 4). These criteria were also not influenced by sex. Notably, consistent with the study of Hou et al. (2023), the level of glutathione was greater in duodenum than in ileum. This difference may be attributed to the role of duodenum being the first site exposed to ingested nutrients, toxin, and potential oxidants, and thus requiring more antioxidant defense.

The present study evaluated the effects of S-ABA supplementation on growth performance and antioxidant status of nursery pigs fed a basal diet formulated to meet or exceed NRC (2012) requirement estimates, including key antioxidant nutrients. Under these conditions, S-ABA supplementation did not produce significant changes in growth performance or antioxidant parameters, likely due to the absence of an oxidative challenge. Despite this, it can be concluded that supplementation of S-ABA in the diet reduced erythrocyte GSSG:GSH indicating improved redox balance. The improvement in the antioxidant status was achieved without detrimental effect on growth performance.

## List of abbreviations

ADFI: average daily feed intake
ADG: average daily gain
BW: body weight
CP: crude protein
EE: ether extract
G:F: gain to feed ratio
GSH: reduced glutathione
GSH+GSSG: total glutathione
GSSG: oxidized glutathione
GSSG:GSH: oxidized to reduced glutathione ratio
MDA: malondialdehyde
PPARγ: Peroxisome proliferator-activated receptor gamma
ROS: reactive oxygen species
S-ABA: S-abscisic acid
SOD: superoxide dismutase
TAC: total antioxidant capacity
TBARS: thiobarbituric acid reactive substances
